# Plant-Dominant Low Protein Diet: A Potential Alternative Dietary Practice for Patients with Chronic Kidney Disease

**DOI:** 10.3390/nu15041002

**Published:** 2023-02-16

**Authors:** Yusuke Sakaguchi, Jun-Ya Kaimori, Yoshitaka Isaka

**Affiliations:** Department of Nephrology, Osaka University Graduate School of Medicine, 2-2 Yamada-oka, Suita 565-0871, Japan

**Keywords:** chronic kidney disease, plant-based diet, low-protein diet, magnesium

## Abstract

Dietary protein restriction has long been a cornerstone of nutritional therapy for patients with chronic kidney diseases (CKD). However, the recommended amount of dietary protein intake is different across guidelines. This is partly because previous randomized controlled trials have reported conflicting results regarding the efficacy of protein restriction in terms of kidney outcomes. Interestingly, a vegetarian, very low protein diet has been shown to reduce the risk of kidney failure among patients with advanced CKD, without increasing the incidence of hyperkalemia. This finding suggests that the source of protein may also influence the kidney outcomes. Furthermore, a plant-dominant low-protein diet (PLADO) has recently been proposed as an alternative dietary therapy for patients with CKD. There are several potential mechanisms by which plant-based diets would benefit patients with CKD. For example, plant-based diets may reduce the production of gut-derived uremic toxins by increasing the intake of fiber, and are useful for correcting metabolic acidosis and hyperphosphatemia. Plant proteins are less likely to induce glomerular hyperfiltration than animal proteins. Furthermore, plant-based diets increase magnesium intake, which may prevent vascular calcification. More evidence is needed to establish the efficacy, safety, and feasibility of PLADO as a new adjunct therapy in real-world patients with CKD.

## 1. Introduction

Metabolites of dietary proteins are mostly excreted by the kidney. These substances accumulate in patients with chronic kidney disease (CKD) due to decreased glomerular filtration and tubular secretion. A myriad of these solutes, such as indoxyl sulfate and p-cresyl sulfate, have been identified as uremic toxins, potentially causing the damage of various organs, including the kidney and cardiovascular system [1]. In addition, several animal studies have shown that a high-protein diet induces glomerular hyperfiltration and enhances the production of pro-inflammatory and pro-fibrotic cytokines [2,3], which can lead to kidney fibrosis and the progression of CKD. A high-protein diet is also involved in the development of several major complications of CKD, such as hyperphosphatemia and metabolic acidosis, which are known risk factors for the progression of CKD and cardiovascular events [4]. Therefore, protein restriction has long been a cornerstone of nutritional therapy for patients with CKD. 

Indeed, several observational studies have shown that a higher dietary protein intake is associated with a rapid decline in kidney function [4,5]. Although there is still a lack of a formal definition of a high-protein diet, individuals with a dietary protein intake of ≥1.2 g/kg per day showed a 2-fold faster decline in estimated glomerular filtration rate (GFR) compared to those with <0.80 g/kg per day in the Alpha Omega Cohort, which included 2255 patients with post-myocardial infarction [6]. While the causality between the high protein intake and rapid decline in kidney function was not proven, this evidence may support the Kidney Disease: Improving Global Outcomes (KDIGO) 2012 CKD guideline that recommends to avoid a protein intake of >1.3 g/kg per day in adults with CKD at risk of progression in order to prevent the deterioration of kidney function [7].

## 2. Recommendation for Protein Restriction in International Guidelines for CKD 

In the KDIGO 2012 CKD guideline, lowering protein intake to 0.8 g/kg per day is recommended for those with a GFR of <30 mL/min per 1.73 m^2^ (CKD stages G4 and G5), irrespective of the presence or absence of diabetes mellitus [7]. This is not a strict protein restriction, considering that the estimated average requirement for protein intake is 0.6 g/kg per day and the recommended daily allowance of protein intake is 0.83 g/kg per day for the healthy adult population [8]. This guideline rather argues that the efficacy of the long-term dietary protein restriction (<0.8 g/kg per day) on the progression of CKD is still controversial. Although meta-analyses of randomized controlled trials (RCTs) have reported that protein restriction reduces the risk of kidney failure, the certainty of the evidence is low owing to a small-study effect and significant study heterogeneity [9,10]. This guideline also concerns protein malnutrition as a result of a strict protein restriction, especially in older CKD patients.

In contrast, the Kidney Disease Outcomes Quality Initiative (KDOQI) 2020 updated clinical practice guideline for nutrition in CKD recommends a low protein diet (LPD) (0.55 to 0.60 g/kg per day) or a very low protein diet (0.28 to 0.43 g/kg per day) supplemented with ketoacid/amino acid analogs (sVLPD) for non-diabetic and metabolically stable patients with pre-dialysis CKD stages G3 to G5 to reduce the risk of death and kidney failure and to improve quality of life [11]. Because the average age of the patients in the pooled analyses conducted in this guideline is <65 years old [12,13,14,15,16], the safety and efficacy of the LPD and sVLPD for older patients are not guaranteed. As mentioned in this guideline, strict protein restriction should be performed under close clinical supervision to avoid nutritional insufficiency and protein energy wasting. Another important issue that has not been fully addressed is how to maintain the long-term patient adherence to these diets. A dietary protein intake of 0.6 to 0.8 g/kg per day is recommended for patients with diabetic kidney disease who are not on dialysis.

## 3. RCTs of the LPD and sVLPD for Patients with CKD

The discrepancy between the two major guidelines described above would be explained by the mixed results of the previous RCTs regarding the efficacy of the protein restriction in patients with CKD.

The Modification of Diet in Renal Disease (MDRD) study 1 is one of the largest studies that compared a usual protein diet (1.3 g/kg per day) with the LPD (0.58 g/kg per day) in 585 patients with a GFR of 25 to 55 mL/min per 1.73 m^2^ [17]. It should be noted that the adherence to the diet protocol was poor; the mean actual protein intake was 1.11 and 0.73 g/kg per day in the usual protein diet and LPD groups, respectively [7]. During the mean follow-up of 2.2 years, there was no significant between-group difference in the change in GFR measured by 125I-iothalamate. Notably, however, there was an initial dip of GFR after the initiation of the LPD, most likely due to the attenuation of glomerular hyperfiltration, followed by a 28% lower GFR decline compared to the usual protein diet group (*p* = 0.009). The latter finding suggests that the LPD could exhibit a reno-protective effect if implemented for a long period. One of the limitations of this study is that angiotensin-converting enzyme inhibitors were used only in 32 to 44% of the study participants. Thus, it is unclear as to whether the LPD improves kidney outcomes over the current standard therapies, including renin-angiotensin-aldosterone (RAS) inhibitors and sodium-glucose cotransporter 2 (SGLT2) inhibitors [18].

In the MDRD study 2, which randomized 255 patients with a GFR of 13 to 24 mL/min per 1.73 m^2^ to the LPD (0.58 g/kg per day) or sVLPD (0.28 g/kg per day supplemented by a keto acid-amino acid supplement), there were no significant differences in the slope of GFR or the rate of kidney failure between the groups [17]. The mean actual protein intake did not meet the target range (0.69 and 0.46 g/kg per day in the LPD and sVLPD groups, respectively), which might have diluted the potential benefit of the sVLPD.

In the long-term follow-up data of the MDRD study 2, in which the median duration of follow-up until death was 10.6 years, the sVLPD group surprisingly showed an 82% higher rate of all-cause mortality compared to the LPD group [19]. This result may partly be attributable to the fact that the average energy intake during follow-up was only approximately 22 kcal/kg per day in both diet groups, suggesting that the strict protein restriction with insufficient energy intake led to protein energy wasting and an increased risk of mortality in the sVLPD group. The finding of this study emphasizes that adequate energy intake (e.g., >30 kcal/kg per day) should be ensured when implementing strict protein restrictions in order to maintain a neutral nitrogen balance.

Cianciaruso et al. reported the result of a 30-month extension study of an initial 18-month RCT comparing an LPD (0.55 g/kg per day without ketoanalogues supplementation) with a moderate protein diet (0.80 g/kg per day) in 423 patients with an eGFR of <30 mL/min per 1.73 m^2^ (baseline mean eGFR, 16 mL/min per 1.73 m^2^) [13]. The average protein intake during the follow-up was 0.73 and 0.90 g/kg per day in the LPD and moderate protein diet groups, respectively. Only 44% of the patients received RAS inhibitors. After the median follow-up of 30 months, no significant difference was observed in the rate of dialysis initiation or death between the two diet groups. The eGFR slopes were also similar. As the adherence to the calorie prescription was verified by a dietitian, the changes in body weight and urinary creatinine excretion, which reflect caloric intake and lean body mass, did not differ between the groups. Collectively, while the LPD did not worsen the nutritional status, it did not improve the kidney outcome and survival compared to the moderate protein diet.

More recently, Bellizzi et al. reported the ERIKA study, a pragmatic RCT in which participants received current tertiary nephrology care [20]. This trial compared the sVLPD (0.30 g/kg per day supplemented with a mixture of essential amino acids and ketoanalogues) with the LPD (0.60 g/kg per day) among 223 patients with CKD stages G4 to G5. Because of the pragmatic study design, this trial was more representative of real-world patients with CKD than the previous ones; for example, approximately 30 to 40% of the participants had diabetes mellitus and cardiovascular complications. More than 75% of the patients received RAS inhibitors. At the same time, the adherence to the study diet was poor; the median protein intake was 0.60 and 0.83 g/kg per day in the sVLPD and LPD groups, respectively. While the amount of urinary protein was significantly reduced by the sVLPD compared to the LPD, there were no significant differences between the groups with respect to the rate of kidney failure, mortality, or cardiovascular events.

Taken together, it remains unclear whether the LPD or sVLPD can slow the progression of CKD and reduce the risk of mortality, especially among those receiving the current standard medical treatment for CKD, such as RAS inhibitors and SGLT2 inhibitors. It is also an important issue to explore a way to improve the adherence to a protein restriction diet. 

## 4. The Protein Sources in the Modern Diet

According to the National Health and Nutrition Examination Survey (NHANES) in 2007 to 2010, the proportions of total protein intake attributable to animal and dairy protein are 46% and 16% in the US, respectively, while plant protein accounted for only 30% both in males and females; chicken and beef were the top two food categories for animal protein [21]. A similar finding was reported from Europe, where approximately 35% of the total protein intake was derived from the plant protein [22]. In contrast, nearly half of the total protein intake is derived from the plant protein in Japan [23]. 

Likewise, in the MDRD study 2, approximately 65% of the dietary protein was from animal sources [19]. Therefore, the findings from the MDRD study may not be extrapolated to diets that are rich in plant protein. It is worth investigating whether different sources of protein have different impacts on kidney outcomes. 

## 5. Does “Vegetarian sVLPD” Improve Kidney Outcomes?

Garneata et al. conducted an open-label, 15-month RCT comparing a vegetarian sVLPD (0.30 g/kg per day supplemented with ketoanalogues of essential amino acids) with a LPD (0.60 g/kg per day including animal proteins) in 207 patients with an eGFR of <30 mL/min per 1.73 m^2^ [15]. One of the characteristics of this trial was that patients who were expected to adhere to the study diet were enrolled. As a result, only 14% of the screened patients were randomized, while a large number of patients were excluded due to difficulty adhering to the study diet. Accordingly, the randomized patients of this trial showed excellent compliance with the diet protocol; the discontinuation rate of the diet during follow-up was only 3%. 

In the vegetarian sVLPD group, serum bicarbonate levels were significantly increased, while serum phosphate, urate, and urea levels were significantly decreased. Moreover, a remarkable reduction was observed in the rate of the primary kidney outcome (initiation of renal replacement therapy and a 50% reduction in eGFR) in the vegetarian sVLPD group compared to that in the LPD group (13% vs. 42%; *p* < 0.001). The number needed to treat for one year to avoid dialysis initiation was only 22.4. There were no significant differences in nutritional parameters between the groups. 

This trial strongly supports the efficacy of vegetarian sVLPD for patients with advanced CKD to reduce the risk of kidney failure although it is difficult to discriminate whether the benefit was derived from the well-conducted strict protein restriction or a vegetarian source of protein. Further studies should address the optimal amount of plant-protein for kidney protection. In addition, a strategy will need to be established to maintain long-term adherence to such a diet, which might be difficult for the majority of CKD patients. 

## 6. A Plant-Based Diet as an Alternative Dietary Practice for Patients with CKD

Recently, plant-based diets, which include high proportions of vegetables, fruits, nuts, whole grains, and legumes, with or without small amounts of meat, fish, and dairy, have received attention due to their health benefits [24]. These include vegan, vegetarian, Mediterranean, and Dietary Approaches to Stop Hypertension (DASH) diets. Plant-based diets are associated with a lower risk of all-cause mortality [25], type 2 diabetes mellitus [26], hypertension [27], and coronary heart disease [28].

Interestingly, in a nationwide prospective cohort study of Chinese adults aged 65 years or older, a higher intake of healthy plant foods (fresh fruits, fresh vegetables, legumes, garlic, nuts, and tea) was associated with a lower rate of mortality, while a higher intake of less healthy plant foods (refined grains, preserved vegetables, and sugar) was associated with a higher rate of mortality [25]. Therefore, future studies should focus on the sources of plant foods, which may provide more relevant clinical implications.

A plant-based diet may also have some favorable effects on the prognosis of patients with CKD [29]. A meta-analysis of 15 cohort studies of 85,437 participants (the average baseline eGFR was 87 mL/min per 1.73 m^2^) showed that healthy dietary patterns such as the DASH and Mediterranean diets were associated with lower odds of incident CKD and albuminuria [30]. More specifically, a higher intake of vegetables and fruits was associated with a lower risk of incident CKD (eGFR <60 mL/min per 1.73 m^2^ or proteinuria) in a community-based prospective cohort study of 9229 Koreans with normal kidney function [31]. Among patients with CKD stages G3 and G4, the NHANES showed that a higher intake of vegetables and fruits was associated with a lower risk of the initiation of renal replacement therapy [32]. Conversely, there is a strong dose-dependent association between red meat intake and the risk of kidney failure, even after adjustment for total protein intake [33]. Notably, substituting one serving of red meat with soy or legumes is associated with a 50.4% reduced risk of kidney failure [33]. These observational studies suggest the benefit of the plant-based diet for preventing the incidence and progression of CKD.

Accordingly, a plant-dominant low-protein diet (PLADO) (dietary protein intake of 0.6 to 0.8 g/kg per day composed of >50% plant-based sources) has been proposed as an alternative dietary therapy for CKD [34,35]. Kalantar-Zadeh mentioned that this dietary regimen can be used safely for the management of CKD, while well-designed, pragmatic randomized controlled trials are warranted to verify its efficacy [34]. 

## 7. Does a Plant-Based Diet Increase the Risk of Hyperkalemia?

Patients with CKD have long been advised to restrict their consumption of vegetables and fruits in order to avoid hyperkalemia. However, it should be recognized that meat, fish, and poultry are also rich in potassium [36]. In addition, potassium bioavailability depends on food sources. Approximately 50 to 60% of potassium in fruits and vegetables is absorbed in the intestine, in contrast to approximately 80% in animal foods [29]. A plant-based diet increases dietary fiber intake, promoting intestinal potassium excretion by increasing stool volume and decreasing transit time. More importantly, potassium additives in processed and ultra-processed foods, widely used in the modern diet, are absorbed nearly 100%. KDIGO emphasizes that salt substitutes, food additives, and preservatives are hidden sources of potassium that can largely increase the total dietary intake (e.g., potassium preservatives in prepared meat may add 300 to 575 mg of potassium per 100 g of intake) [36]. As a result, there is only a weak association between dietary potassium intake and serum potassium levels both in non-dialysis CKD patients and those undergoing hemodialysis [37,38,39]. Therefore, a plant-based diet is not necessarily considered to increase the risk of hyperkalemia. Nevertheless, most educational materials for patients with CKD primarily recommend restricting fruits and vegetables, while only a few materials focus on potassium additives in ultra-processed foods [40].

Indeed, in the RCT by Garneata et al., the vegetarian sVLPD did not significantly increase serum potassium levels, even in patients with an eGFR of <30 mL/min per 1.73 m^2^ [15]. This may be partly attributable to the remarkable amelioration of metabolic acidosis by the study diet. In a single arm study of 13 patients with CKD stages G3 and G4, Moorthi et al. also reported that a 70% plant-protein-based diet for 4 weeks did not alter serum potassium levels [41]. These findings support the notion that a plant-based diet could be widely applicable to patients with CKD without much concern about the risk of hyperkalemia.

Patients undergoing hemodialysis have also been recommended to restrict vegetables and fruits to avoid hyperkalemia. Although intervention studies examining the plant-based diets in hemodialysis patients are lacking, Gonzalez-Ortiz et al. reported that a higher adherence to a healthy plant-based diet (i.e., a higher intake of cereals, fruits, and vegetables and a lower intake of sugar and animal foods) estimated from 3-day food records was not associated with hyperkalemia among 150 patients undergoing hemodialysis [42]. The amount of potassium intake was also not significantly different across patients with low, moderate, and high adherence to the diet, whereas the amount of fiber was significantly higher in those with high adherence [42]. Therefore, a plant-based diet could also be a choice for hemodialysis patients, although more evidence is required to assess its safety and feasibility in this population. 

## 8. Benefits of a Plant-Based Diet for Patients with CKD

**Figure 1:**
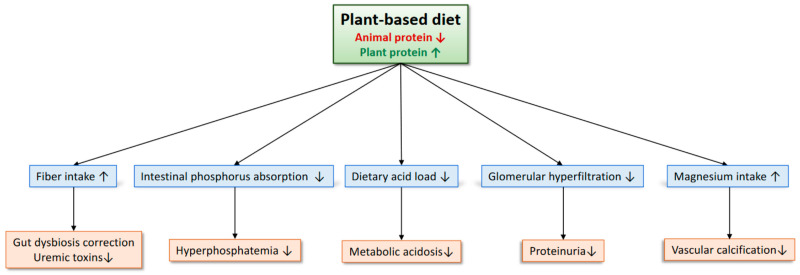
**Potential benefits of plant-based diet for patients with CKD**. Plant-based diets increase fiber intake, which may improve gut dysbiosis and reduce the production of gut-derived uremic toxins. Plant phosphorus is less absorbable than animal phosphorus because it is bound to phytates, which are difficult for humans to digest. Thus, plant-based diets contribute to reducing the phosphorus burden. Alkali-rich plant foods reduce dietary acid load and correct metabolic acidosis. Plant proteins are less likely to induce glomerular hyperfiltration compared to animal proteins. Finally, plant-based diets increase magnesium intake, which may prevent vascular calcification. ↑: increase, ↓: decrease.

There are several potential mechanisms by which patients with CKD would benefit from a plant-based diet (Figure 1).

(1) Uremic toxins are involved in the progression of CKD and cardiovascular events [1]. Patients with advanced CKD often develop metabolic acidosis with elevated anion gap due to the accumulation of uremic anions, which has recently been shown to be associated with a higher risk of CKD progression and cardiovascular events [43,44]. Reducing the production of uremic toxins, therefore, may be useful to improve clinical outcomes of patients with CKD. Many of the protein-bound uremic toxins, such as indoxyl sulphate and p-cresyl sulfate, are derived from the by-products of aromatic amino acid breakdown by the gut microbiome. A plant-based diet may reduce gut-derived uremic toxins by increasing fiber intake and modulating the intestinal microbiota. In an RCT of 40 patients undergoing hemodialysis, an increased intake of dietary fiber for 6 weeks led to a 29% reduction in a free plasma level of indoxyl sulfate [45]. A meta-analysis of RCTs also reported a significant reduction in various uremic solutes by dietary fiber, although evidence in non-dialysis patients with CKD is scarce [46].
(2) Metabolic acidosis is a risk factor for the progression of CKD, especially when pH is low [47]. Acid load is detrimental to the kidney via activation of the angiotensin-aldosterone system, endothelin 1, and the complement pathway [48]. Although sodium bicarbonate has been used to treat metabolic acidosis, it may become a cause of sodium retention and an elevation in blood pressure. Increasing alkali-producing plant foods and decreasing acid-producing animal foods are another efficacious way in correcting metabolic acidosis. Goraya et al. reported that 3 years of dietary acid reduction with fruits and vegetables increased plasma total CO2 levels, reduced urinary angiotensinogen, and delayed the deterioration of GFR in patients with CKD stage G3 without increasing plasma potassium levels [49]. Fruits and vegetables may be preferable to sodium bicarbonate as an alkali therapy for patients with CKD because fruits and vegetables significantly reduce blood pressure levels compared to sodium bicarbonate, presumably by decreasing the sodium load [49]. Further evidence is needed to examine the feasibility of this dietary therapy in the real-world clinical setting of patients with CKD.
(3) Elevated serum phosphate levels lead to vascular calcification and are associated with an increased risk of cardiovascular events and mortality in patients with CKD, although the benefits of phosphate-lowering therapies on clinical outcomes remain uncertain [50]. Because plant phosphorus is bound to phytates, which are difficult for humans to digest, it is much less bioavailable than animal phosphorus and inorganic phosphorus in food additives. Therefore, a plant-based diet can reduce the phosphorus burden. In fact, a 70% plant-protein-based diet for 4 weeks significantly decreases urinary phosphorus excretion and serum fibroblast growth factor 23 levels [41]. In contrast to phosphate binders, which may increase the occurrence of nausea and constipation, a plant-based diet would be helpful in reducing such adverse gastrointestinal effects by increasing the dietary intake of fiber.
(4) Plant proteins are less likely to induce glomerular hyperfiltration compared to animal proteins. Kontessis et al. have reported that soy protein neither increased GFR nor renal plasma flow as animal protein did among healthy individuals [51]. Additionally, urinary albumin clearance was significantly lower after the ingestion of soy protein compared to that of animal protein. Therefore, plant proteins may be advantageous over animal proteins to prevent the elevation in glomerular pressure. Further studies should clarify whether this beneficial effect of soy protein can be observed in patients with CKD who already receive RAS inhibitors and SGLT2 inhibitors.
(5) A plant-based diet can increase the intake of magnesium [52]. Experimental evidence shows that magnesium inhibits calcification of vascular smooth muscle cells induced by phosphate [53]. A high magnesium diet prevented aortic calcification in animal models of CKD [53]. In an RCT of patients with CKD stages G3 and G4, an oral magnesium supplementation significantly retarded the progression of coronary artery calcification [54]. Magnesium might also improve the prognosis of patients with CKD, as described below [55,56].

## 9. Magnesium and Clinical Outcomes in CKD

### 9.1. Prevalence and Causes of Hypomagnesemia in CKD

Hypomagnesemia is not rare in patients with CKD. In a cross-sectional study of 5126 patients with pre-dialysis CKD, the prevalence of hypomagnesemia (serum magnesium levels of <1.8 mg/dL) was approximately 15% [57].

There are several causes of hypomagnesemia in CKD patients. For example, dietary restriction of potassium limits the intake of magnesium. Diuretics and diabetes mellitus are known to enhance urinary magnesium excretion. Furthermore, an animal study has shown that tubular injury induces urinary magnesium wasting [58]. 

### 9.2. Hypomagnesemia and the Risk of CKD Progression

Hypomagnesemia is associated with an increased risk of kidney failure in diabetic kidney disease [59]. This association may be partly explained by the fact that hypomagnesemia represents a surrogate for tubular injury. However, magnesium may be directly involved in CKD progression. In an animal study, a low-magnesium diet aggravates tubular injury and kidney fibrosis induced by a high-phosphate diet [60]. Moreover, magnesium attenuates phosphate-induced cell death, mitochondrial dysfunction, and inflammation of tubular cells [61]. Therefore, magnesium may be useful in preventing phosphate-induced kidney injury. 

### 9.3. Magnesium as a Calcification Inhibitor

Magnesium has a strong physicochemical potential to inhibit calcium–phosphate crystallization. Indeed, in vitro and animal studies showed that magnesium suppresses vascular calcification [53,62,63]. In an RCT of 96 patients with CKD stages G3 and G4, oral magnesium oxide retarded the progression of coronary artery calcification [54]. An RCT of 72 hemodialysis patients also showed the superiority of magnesium carbonate over calcium carbonate in preventing arterial calcification progression [64].

Magnesium is known to antagonize the maturation of calciprotein particles (CPPs) that are involved in vascular calcification [56]. A high-magnesium dialysate has been reported to improve T50, a marker of inhibitory capacity of serum against the maturation of CPPs [65].

### 9.4. Magnesium and Clinical Outcomes in Hemodialysis Patients

Lower serum magnesium levels were associated with a higher risk of mortality in hemodialysis patients [66]. Those with mild hypermagnesemia (pre-dialysis serum magnesium levels of 2.7 to 3.0 mg/dL) showed the best survival. 

Mild hypermagnesemia may be suitable for hemodialysis patients to maintain ionized magnesium levels. The ionized-to-total magnesium ratio is low in these patients, probably due to increased plasma anions such as phosphate, which form a complex with magnesium ions [67]. Therefore, mild hypermagnesemia is favorable for maintaining ionized magnesium levels, especially in patients with hyperphosphatemia. 

It may be a concern that excess magnesium may impede bone mineralization. However, the risk of hip fracture was not elevated in patients with mild hypermagnesemia [68].

**Figure 2:**
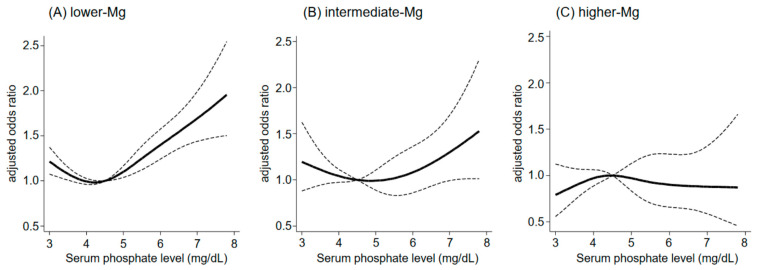
**Serum magnesium levels modify the association between serum phosphate levels and cardiovascular death in hemodialysis patients.** (**A**) High serum phosphate levels are associated with an increased risk of cardiovascular death in those with serum magnesium levels <2.7 mg/dL. However, this association is attenuated in those with serum magnesium levels 2.7–3.0 mg/dL (**B**) and 3.1 mg/dL or greater (**C**). The dashed lines represent 95% confidence intervals. Cited from Ref. [69]; Abbreviation: Mg, magnesium.

Interestingly, there is a significant interaction between magnesium and phosphate in terms of cardiovascular death [69]. Higher serum phosphate levels were associated with an increased risk of cardiovascular death when serum magnesium levels were <2.7 mg/dL, whereas this association was attenuated with the increase in serum magnesium levels (Figure 2) [69]. Therefore, magnesium may be particularly useful for alleviating the risk of cardiovascular events related to hyperphosphatemia. 

## 10. Conclusions

According to the evidence discussed in this review, PLADO may serve as a new adjunct therapy to delay the progression of CKD. Additionally, this diet can be efficacious in managing complications of CKD, including metabolic acidosis, hyperphosphatemia, accumulation of uremic toxins, and glomerular hyperfiltration. Furthermore, increased magnesium consumption may prevent vascular calcification and phosphate-induced kidney injury. Current evidence does not suggest the risk of hyperkalemia under a plant-based diet in patients with advanced CKD, although close monitoring is recommended in the clinical setting. Further evidence is required to establish the efficacy, safety, and feasibility of this diet in real-world patients with CKD.

## Data Availability

Not applicable.
